# Comprehensive Molecular Analyses of a Six-Gene Signature for Predicting Late Recurrence of Hepatocellular Carcinoma

**DOI:** 10.3389/fonc.2021.732447

**Published:** 2021-09-09

**Authors:** Yuyuan Zhang, Zaoqu Liu, Xin Li, Long Liu, Libo Wang, Xinwei Han, Zhen Li

**Affiliations:** ^1^Department of Interventional Radiology, The First Affiliated Hospital of Zhengzhou University, Zhengzhou, China; ^2^Interventional Institute of Zhengzhou University, Zhengzhou, China; ^3^Interventional Treatment and Clinical Research Center of Henan Province, Zhengzhou, China; ^4^Department of Hepatobiliary and Pancreatic Surgery, The First Affiliated Hospital of Zhengzhou University, Zhengzhou, China

**Keywords:** stages I–III hepatocellular carcinoma, LASSO, gene signaling, genomic alterations, late recurrence

## Abstract

A larger number of patients with stages I–III hepatocellular carcinoma (HCC) experience late recurrence (LR) after surgery. We sought to develop a novel tool to stratify patients with different LR risk for tailoring decision-making for postoperative recurrence surveillance and therapy modalities. We retrospectively enrolled two independent public cohorts and 103 HCC tissues. Using LASSO logical analysis, a six-gene model was developed in the The Cancer Genome Atlas liver hepatocellular carcinoma (TCGA-LIHC) and independently validated in GSE76427. Further experimental validation using qRT-PCR assays was performed to ensure the robustness and clinical feasible of this signature. We developed a novel LR-related signature consisting of six genes. This signature was validated to be significantly associated with dismal recurrence-free survival in three cohorts TCGA-LIHC, GSE76427, and qPCR assays [HR: 2.007 (1.200–3.357), *p* = 0.008; HR: 2.171 (1.068, 4.412), *p*-value = 0.032; HR: 3.383 (2.100, 5.450), *p*-value <0.001]. More importantly, this signature displayed robust discrimination in predicting the LR risk, with AUCs being 0.73 (TCGA-LIHC), 0.93 (GSE76427), and 0.85 (in-house cohort). Furthermore, we deciphered the specific landscape of molecular alterations among patients in nonrecurrence (NR) and LR group to analyze the mechanism contributing to LR. For high-risk group, we also identified several potential drugs with specific sensitivity to high- and low-risk groups, which is vital to improve prognosis of LR-HCC after surgery. We discovered and experimentally validated a novel gene signature with powerful performance for identifying patients at high LR risk in stages I–III HCC.

## Introduction

Hepatocellular carcinoma (HCC) is a highly malignant cancer with a poor prognosis (1). Currently, liver resection and interventional treatment have been the mainstream curative treatment of stages I–III patients with HCC, while the high recurrence rate remains the major obstacle to improve long-term survival, with almost 70% of the patients experiencing recurrence after surgery within 5 years (2, 3). Relative to the time from surgery to initial recurrence, HCC recurrence is typically divided into early and late recurrence (LR), which has generally been defined using 2 years as a cutoff value (4). In clinical practice, many patients who were alive and free of tumor recurrence at 2 years after curative liver resection of HCC do not take surveillance regularly, and therefore may lose the chance to undergo curative treatment when symptoms develop. Therefore, it is necessary to predict patients who may be susceptible to LR and further provide optimized strategies of recurrence surveillance and treatment.

As reported, patterns and extent of initial recurrence were different among patients with early and late recurrence (5). The LR is probably related to the evolution of the underlying chronic liver diseases, which was generally considered a *de novo* tumor with different biologic behaviors compared with early recurrence (6). It is researched that the relationship between cirrhosis and LR has clinical “face validity,” as chronic hepatitis inflammation and fibrosis accelerated HCC development by generating a carcinogenic microenvironment in the liver, known as the “field effect” (7). Hence, it is necessary to decipher the genomic landscape among the patients with LR which may contribute to tumor development and progression. Furthermore, we hope to translate this knowledge into new biomarkers and targets in order to have an impact on decision-making of surveillance and treatment, and ultimately improve the clinical outcomes of HCC patients.

At present, clinicians generally choose a rational treatment strategy based on the Tumor-Node-Metastasis (TNM) staging system. However, HCC patients with the same TNM stage tend to have distinct prognosis and thus need more individualized management strategies. Until recently, there have been a few published studies on LR of HCC and are mostly limited in the clinical characteristics. For example, a study from the Eastern Hepatobiliary Surgery Hospital investigated the risk factors of LR after liver resection for hepatitis B virus (HBV)-associated HCC. In this study, Wang et al. found that the rate of recurrence increased with a peak at 1–2 and 4–5 years after surgery (approximately 23% and 35%/year, respectively) and concluded that male, liver cirrhosis, and a high preoperative HBV-DNA load were associated with LR (8). Most past studies did not, however, elucidate details regarding the accurate prediction of LR, as well as the rational intervention for the high-risk patients for LR (4, 7, 8). Nowadays, we can easily obtain a large scale of genes for downstream analysis. With the help of machine learning, such as the least absolute shrinkage and selection operator (LASSO) algorithm (9), it is possible to identify the most important elements based on the expression profiles of global genes and fit a model with strong generalization performance.

To the best of our knowledge, we firstly explored and delineated the genomic landscape of LR-HCC and performed a comprehensive biomarker discovery, as well as validation work to develop a LR-related signature for predicting the LR of patients with stages I–III HCC. Furthermore, we used 103 frozen tissue samples with qRT-PCR data for experimental verification to prove the stability and reliability of the model. Herein, we reported a novel six-gene signature, which not only offered stable and excellent accuracy in identifying patients at high LR risk but also can be readily translated into clinical practice due to the simplicity and inexpensiveness of PCR-based assays. Overall, we believe the LR-related signature offers an attractive platform for evaluating LR risk of patients with stage I-III HCC and is helpful to inform rational strategies of surveillance after liver resection, as well as decision making about treatment options for LR-HCC.

## Methods

### Data Acquisition and Arrangement

The liver hepatocellular carcinoma (LIHC) transcriptome profiles with clinical data were obtained from The Cancer Genome Atlas (TCGA, https://portal.gdc.cancer.gov/) and the chip-array profiles with clinical data were downloaded from Gene Expression Omnibus (GEO, https://www.ncbi.nlm.nih.gov/geo/). Samples were enrolled using the following criteria for further analysis: (1) primary hepatocellular carcinoma; (2) AJCC stages I–III; and (3) with recurrence data. Eventually, 73 patients met the criteria in the TCGA-LIHC, including 26 LR patients and 47 NR patients, and 21 patients met the criteria in the GSE76427, including nine LR patients and 12 NR patients. RNA-seq data (FPKM normalized) of TCGA-LIHC were transformed to log_2_ (transcripts per kilobase of exon model per million mapped reads (TPM) +1). Subsequently, we prepared the corresponding somatic mutation data from the datasets and implemented the maftools package which provides various functions to perform feature-rich customizable visualizations. The corresponding copy number variation (CNV) data of the TCGA was analyzed and downloaded from the cBioPortal datasets (http://www.cbioportal.org/). Microarray raw data of GSE76427 obtained from the GEO database were further processed and normalized using lumi R package.

### Identification of Significantly LR-Related Genes in HCC

To search the genes significantly related to LR in HCC, we initially filtered LR-related genes in TCGA-LIHC and GSE76427 using DEseq2 and limma R packages, separately, and the adjusted *p*-value <0.05 was adopted for further analysis. The co-upregulated and co-downregulated genes of the two expression profiles were then determined with VennDiagram R package. Univariate Cox regression analysis or Kaplan-Meier survival analysis were conducted to screen genes that significantly correlated with RFS.

### Development of the LR-Related Signature in HCC

The least absolute shrinkage and selection operator (LASSO) logistic regression analysis was used to build the LR-related model in HCC using glmnet packages. By tenfold crossvalidation, the optimal lambda was generated when the partial likelihood de*via*nce reached the minimum value (*λ* = 0.012). Based on the optimal lambda, genes with nonzero coefficients were selected to establish the prediction model. The risk score for each patient was calculated with the LASSO model weighting coefficient as follows:

$$\text{Risk score}=\sum_{i=1}^{n}\text{Coef}(i)\times \text{Exp}(i)$$

where *n* is the number of key genes, Coef(*i*) is the LASSO coefficient of gene *i*, and Exp(*i*) is the expression of gene *i*.

### Human Tissue Specimens and qRT-PCR Analysis

We collected a total of 103 frozen surgically resected HCC tissues with AJCC stages I–III at The First Affiliated Hospital of Zhengzhou University. Detailed baseline data of HCC patients are displayed in **Table 1**. Total RNA was isolated from HCC tissues using RNAiso Plus reagent (Takara, Dalian, China) according to the manufacturer’s instructions. RNA quality was evaluated using a NanoDrop One C (Waltham, MA, USA), and RNA integrity was assessed using agarose gel electrophoresis. An aliquot of 1 µg of total RNA was reverse-transcribed into complementary DNA (cDNA) according to the manufacturer’s protocol using the mRNA reverse transcription Kit (TaKaRa BIO, Shiga, Japan). All cDNA samples were prepared for qRT-PCR. This project was approved by the Ethics Committee Board of The First Affiliated Hospital of Zhengzhou University. In the qRT-PCR analysis, the enrolled six genes in the model were detected. qRT-PCR was performed using SYBR Assay I Low ROX (Eurogentec, USA) and SYBR^®^ Green PCR Master Mix (Yeason, Shanghai, China). The 2^−ΔΔCt^ method was used to calculate the relative levels of gene and mRNA expression, and then log_2_ transformed for subsequent analysis. The primer sequences of the included six genes and *GAPDH* are shown in **Table S4**.

**Table 1:** Detail clinical data of qRT-PCR data from 103 samples.

	NR (N=61)	LR (N=42)	Pval	ALL (N=103)
Age > 60:			1	
Yes	12 (19.7%)	8 (19.0%)		20 (19.4%)
No	49 (80.3%)	34 (81.0%)		83 (80.6%)
Sex:			0.008	
Male	38 (62.3%)	37 (88.1%)		75 (72.8%)
Female	23 (37.7%)	5 (11.9%)		28 (27.2%)
Cirrhosis:			0.028	
Yes	41 (67.2%)	37 (88.1%)		78 (75.7%)
No	20 (32.8%)	5 (11.9%)		25 (24.3%)
Preoperative AFP level >400 μg/L:			0.94	
Yes	22 (36.1%)	14 (33.3%)		36 (35.0%)
No	39 (63.9%)	28 (66.7%)		67 (65.0%)
Stage:			0.95	
I	37 (60.7%)	27 (64.3%)		64 (62.1%)
II	19 (31.1%)	12 (28.6%)		31 (30.1%)
III	5 (8.20%)	3 (7.14%)		8 (7.77%)
Tumor size >5.0 cm:			0.845	
Yes	18 (29.5%)	14 (33.3%)		32 (31.1%)
No	43 (70.5%)	28 (66.7%)		71 (68.9%)
Multiple tumors:			0.454	
Yes	11 (18.0%)	11 (26.2%)		22 (21.4%)
No	50 (82.0%)	31 (73.8%)		81 (78.6%)
Macrovascular invasion:			0.698	
Yes	5 (8.20%)	2 (4.76%)		7 (6.80%)
No	56 (91.8%)	40 (95.2%)		96 (93.2%)
Microvascular invasion:				
Yes	23 (37.7%)	14 (33.3%)		37 (35.9%)
No	38 (62.3%)	28 (66.7%)		66 (64.1%)

### The Function and Pathway Enrichment Analysis in LR-HCC

Moreover, we evaluated the functions of the identified LR-related genes using Gene Ontology (GO) function analysis [biological processes (BP), molecular functions (MF), and cellular components (CC)] and Kyoto Encyclopedia of Genes and Genomes (KEGG) through the clusterProfiler package. To further reveal the latent functions underlying the different risk groups, the gene‐set enrichment analysis (GSEA) algorithm was carried out to identify enriched dramatical terms correlated with KEGG pathway and GO. We set the number of random permutations as 1,000 to generate a normalized enrichment score (NES) and false-discovery rate (FDR) *q*-value. The terms with NES >2 and FDR *q*-value <0.01 were deemed as strikingly enriched.

### Immune Cell Infiltration Assessment

Subsequently, we used CIBERSORT (10) algorithm to estimate abundance of 22 immune cells in the tumor immune microenvironment (TME). xCell (11) algorithm was also utilized to infer the abundance scores of 64 immune and stromal cells in two groups. As reported, immune-regulating factors such as co-stimulators, co-inhibitors, and other factors, which exert a role of antitumor or promoting tumor are involved in regulating the functions of immune cells. Therefore, a total of 27 immune regulators were assembled in our study, and the differences between two groups were investigated to elucidate the immune status.

### Genomic Landscape and Chemotherapeutic Response Prediction in Two Groups

The online dataset cBioPortal was utilized to identify the significantly mutated genes (SMGs) and significantly altered segments (SAEs) for the two risk groups of HCC. Genes with the top 20 mutation frequency were defined as SMGs, and fragments with top 20 alteration frequency were defined as SAEs. A previous work has proposed a ridge regression model to evaluate the imputed response to 138 chemotherapeutic agents based on pharmacogenomics and gene expression data. The pRRophetic (12) R package was conducted to perform the prediction process. The half‐maximal inhibitory concentration (IC_50_) was utilized to quantify drug sensitivity and the lower the IC_50_, the higher the sensitivity. In order to better display the difference of potential chemotherapeutic agents between two groups, we labeled the sensitivity of two groups as “high sensitivity” and “low sensitivity,” according to the criteria we established before (13).

### Statistical Analysis

All data processing, statistical analysis, and plotting were conducted in R 4.0.2 software. Continuous variables were compared between two groups through the Wilcoxon rank-sum test or *t*-test. Fisher’s exact test or Pearson’s Chi-squared test was applied to compare categorical variables. All *p*-values were two sided, with *p*-value <0.05 considered to be of statistical significance.

## Results

### Mutation Landscape of LR-HCC

**Figure 1:**
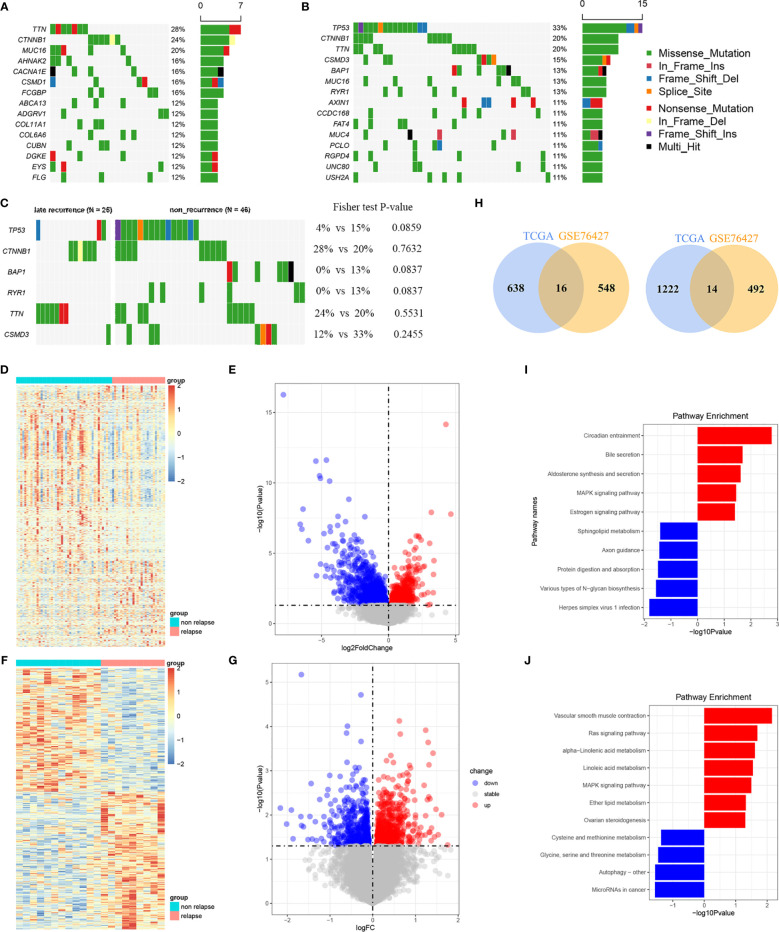
Analyses of somatic mutation profiles in HCC samples and filtration of LR-related genes. **(A, B)** Waterfall plot of detailed mutation information for top 10 genes in the 23 and 46 HCC patients with LR **(A)** and 46 with NR **(B)**, respectively. **(C)** Waterfall plot of the different mutation genes between LR- and NR-HCC. **(D, E)** The heatmap **(D)** and volcano plots **(E)** of LR genes in the TCGA-LIHC. **(F, G)** The heatmap **(F)** and volcano plots **(G)** of LR genes in the GSE76427 cohort. **(H)** The Venn diagrams of the down and up overlap genes. **(I)** The KEGG enrichment analysis between LR- and NR-HCC in the TCGA cohort. **(J)** The KEGG enrich analysis of the common LR-related genes.

Mutation landscape in LR- and NR-HCC patients was firstly demonstrated in our study. The waterfall plot exhibited the detailed mutation information in each sample, with various color annotations to distinguish different mutation types (**Figure 1A**). The top 10 mutation genes in the LR group are *TTN* (28%), *CTNNB1* (24%), *MUC16* (20%), *AHNAK2* (16%), *CACNA1E* (16%), *CSMD1* (16%), *FCGBP* (16%), *ABCA13* (12%), *ADGRV1* (12%), and *COL11A1* (12%) (**Figure 1A**), which were different from the NR-HCC, *TP53* (33%), *CTNNB1* (20%), *TTN* (20%), *CSMD3* (15%), *BAP1* (13%), *MUC16* (13%), *RYR1* (13%), *AXIN1* (11%), *CCDC168* (11%), and *FAT4* (11%) (**Figure 1B**). Furthermore, we compared the frequency of somatic mutations between LR- and NR-HCC patients from TCGA-LIHC. Intriguingly, *TP53*, *RYR1*, and *BAP1* mutations were noted to occur more in NR-HCC patients instead of LR-HCC patients (**Figure 1C**) though statistic difference in the two groups is not significant. This might partially be due to the limited sample size. Tumor mutation burden (TMB) exhibited a similar trend (**Figure S1B**). According to further analysis, in the LR-HCC group, missense mutations, single-nucleotide polymorphism (SNP), and C>T transition accounted for majority of different classification categories, respectively. The median value of mutations in the samples was 81.76, and the maximum was 322 (**Figure S1A**). A previous study reported that the co-occurrence of somatic mutations is commonly noted in tumorigenesis which displays variant roles to impact prognosis and treatment. In the LR-HCC group, we identified some co-occurrence genes, such as *FLG2* and *HRNR*, *CACNA1E*, and *COL5A6* (**Figure S1C**) which have not been reported before. Therefore, HCC patients after surgery may be characterize by the presence/absence of concurrent genomic aberrations. Overall, LR-HCC group performed different distributions and patterns of genomic alterations which may lead to a distinct clinicopathological progression.

### Filtration and Enrichment Analysis of the SLRGS

A total of 1,890 LR-related genes in TCGA-LIHC were screened by DEseq2 (1236 upregulated genes and 654 downregulated genes). These genes were presented in the heatmap and volcano plots (**Figures 1D, E**). Functional enrichment analysis was performed using GO and KEGG analyses to further explore the involved biological functions of these LR-related genes. The top MF of the 1,890 LR-related genes included enzyme activity and RNA-binding related MFs such as acting on NAD(P)H, oxidoreductase activity, structural constituent of ribosome. In terms of BP, majority of these genes were involved in protein translation and targeting transportation-related BPs including mitochondrial translation, SRP-dependent co-translational protein targeting the membrane, and nuclear division. These genes are the main cell components of ribosome subunit and mitochondrial protein complex (**Figure S1D**). The KEGG analysis suggested that these LR-related genes were mainly involved in pathways related to cancer- and metabolism-related pathways, like MAPK signaling pathway, estrogen signaling pathway, bile secretion, and aldosterone synthesis and secretion (**Figure 1I**). GSEA analysis using hallmark pathway database validated the results, and some cancer- and metabolism-related pathways were enriched (**Figure S1E**). Subsequently, LR-related genes were also screened by limma in GSE76427 (totally 1,070, including 506 upregulated genes and 564 downregulated genes) (**Figures 1F, G**). Then, taking the intersection of upregulated and downregulated genes both in TCGA-LIHC and GSE76427, a total of 14 upregulated and 16 downregulated genes were determined **(** **Figure 1H**). KEGG of these 30 overlapping genes were also enriched in the cancer and metabolism-related pathway mentioned above, comprising Ras signaling pathway, MAPK signaling pathway, alpha-linolenic acid pathway, and vascular smooth muscle contraction (**Figure 1J**).

### Construction and Validation of the LR-Related Signature

**Figure 2:**
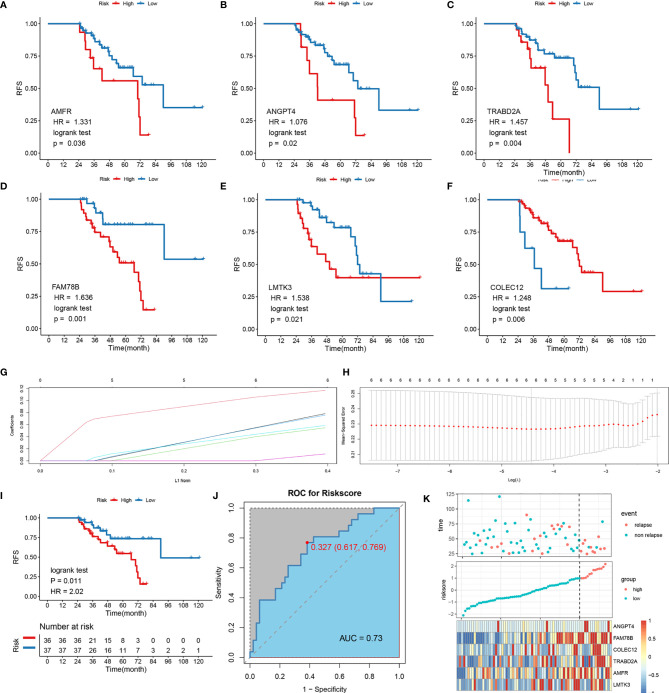
Survival analysis of the six SLRGs and development of the LR-related signature in the TCGA-LIHC cohort. **(A–F)** The Kaplan–Meier analysis of the six SLRGs. The six SLRGs were *ANGPT4*, *AMFR*, *COLEC12*, *FAM78B*, *LMTK3*, and *TRABD2A*. **(G**, **H)** The results of the LASSO regression. **(I)** Kaplan–Meier analysis. **(J)** The ROC curve of the model. **(K)** The distribution of risk score, recurrence status, and gene expression panel.

Thirty overlapping genes associated with LR were further screened using univariate Cox regression analysis and Kaplan-Meier analysis in both the TCGA-LIHC and GSE76427 cohort. Eventually, a total of seven and 11 SRLGs were identified in TCGA-LIHC and GSE76427 cohort, respectively. In aggregate, six overlapping SRLGs were identified, including *ANGPT4*, *AMFR*, *COLEC12*, *FAM78B*, *LMTK3*, and *TRABD2A*. Based on the optimal cutoff point determined by survminer package, Kaplan-Meier analysis of these six genes indicated an obvious distinction in disease progression time in the TCGA train cohort (**Figures 2A–F**), and similar trend was observed in the external validation cohort GSE76427 (**Figures S2A, B**). Through the LASSO logical regression, we further minimized over-fitting and narrowed the number of genes related with RFS (**Figures 2G, H**). Meanwhile, an optimal prognostic signature based on SRLGs was identified, and the formula for our model was risk score = 0.356 + 0.06 × Exp ANGPT4 + 0.110 × Exp FAM78B + 0.046 × Exp COLEC12 + 0.063 × Exp TRABD2A + 0.049 × Exp AMFR + 0.004 × Exp LMTK3. In addition, the risk score of each patient in our study cohort was calculated.

### Survival Outcomes and Multivariate Examination

The optimal risk score cutoff was used to stratify the patients into high-risk and low-risk groups. Obviously, patients in the low-risk group had a lower rate of relapse (low-risk **vs.*.* high-risk: 26.7% **vs.*.* 76.9%) and longer remission than those in the high-risk group in TCGA-LIHC (*p*-value = 0.011; **Figure 2I**). To estimate the power of the signature, the ROC analysis was performed (AUC = 0.73; **Figure 2J**). This finding was validated in the external validation data, GSE76427. Kaplan-Meier survival analysis showed a significant difference in the RFS between the two groups. The model perfectly distinguished LR from NR (high-risk **vs.*.* low-risk: 80% **vs.*.* 9%; **Figure S2G**) with a high precision of AUC = 0.94 (**Figure S2H**). The relative gene expression levels and distribution of the six significantly LR-related genes (SLRGs) are shown in **Figures 2K, I**. The predictive value of the risk score was compared with the following clinical indicators: age, sex, stage, grade, creatinine, and prothrombin time (PT). Univariate Cox regression analysis demonstrated that HR of risk score is 2.020 [1.339–3.047] (*p*-value <0.001) (**Figure S2J**) and multivariate Cox regression analysis determined that the risk score was an independent risk factor for LR of HCC [HR: 2.007 (1.200–3.357)] (**Figure S2K**). In line with the RFS, high-risk group was inclined to possess worse clinical outcomes, such as advanced clinical stage (**Table S2**). The risk score was also validated as an independent risk factor in the external cohort, GSE76427 (**Table S3**). These results suggested that the six-gene signature was an independent prognostic factor for LR-HCC.

### Validation of LR-Related Signature in a Clinical In-House Cohort

**Figure 3:**
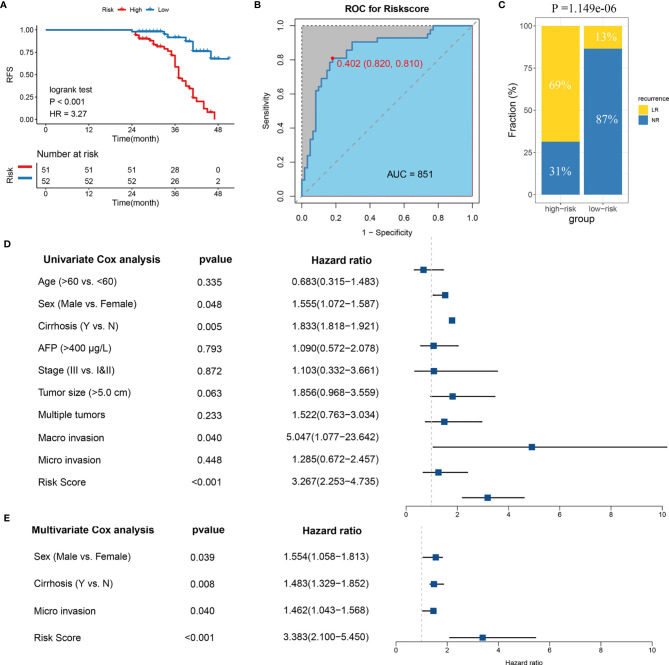
Validation of the model in clinical in-house cohort. **(A)** Kaplan-Meier curves of RFS according to LR-related model. **(B)** ROC analysis for predicting LR. **(C)** Comparison of LR rates between the high- and low-risk groups. **(D)** Univariate Cox regression analysis of risk score. **(E)** Multivariate Cox regression analysis of risk score.

In order to verify the power of our six-gene model into a clinically translatable risk-stratification assay, we further performed qRT-PCR assays for these genes in a clinical cohort containing 103 HCC patients. Expression heatmap of the six SLRGs, distribution of SLRGs, and recurrent status of each patient are illustrated in **Figure S3G**. Consistent with our discovery *in silico* validation cohorts, patients with high score have a significantly dismal RFS (*p*-value <0.001; **Figure 3A**). The six-gene model perfectly distinguished LR- from NR-HCC (high-risk **vs.*.* low-risk: 69% **vs.*.* 13%; **Figure 3C**), with a high precision AUC = 0.851 (**Figure 3B**). Univariate (HR: 3.383 [2.253-4.735], P-value <0.001; **Figure 3D**) and multivariate (HR: 3.383 [2.100-5.450], P-value <0.001; **Figure 3E**). Cox regression analysis revealed that the 6-gene signature remained the statistical significance, after adjusting for potential confounding factors (including sex, cirrhosis, and microvascular invasion). Collectively, the results from a clinical in-house cohort supported that our discovery and *in silico* validation cohort findings, which validated and confirmed that our six-gene model was quite robust and can serve as an independent predictor of LR in stages I–III HCC.

### Somatic Mutation and CNV Landscape of High- and Low-Risk Groups

**Figure 4:**
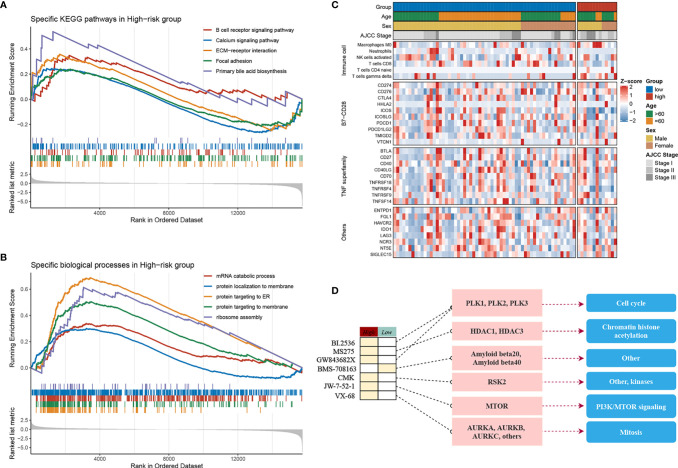
Molecular landscape, immune landscape, and assessment of chemotherapy. **(A, B)** The GSEA analysis of the high- and low-risk groups. **(A)** The KEGG pathway enrichment analysis and **(B)** The biology process enrichment analysis. **(C)** The heatmap of the clinical- and immune-related molecular landscape. From the top to the end, there are five models, including clinical characteristics, immune cells, B7-CD28, TNF superfamily, and other immune-related molecular landscapes. **(D)** Molecular regulatory mechanism of the seven potential antitumour drugs. Left, the drug names and the level of sensitivity in each group; middle, the drug-targeted molecules; right, the drug-targeted pathways.

The samples were allocated into high‐ and low‐risk groups to distinguish their potential functions and elucidate the significant survival differences using GSEA. Immune- and metabolism-related pathways were enriched in the high-risk group, such as, B-cell receptor signaling pathway, focal adhesion, and primary bile acid biosynthesis (**Figure 4A**). Protein synthesis and transfer-related BPs such as mRNA catabolic process, protein localization to membrane, protein targeting ER, and ribosome assembly are the top BPs in the high-risk group (**Figure 4B**). The results showed that not only was there a difference in enriched functions between high- and low-risk groups but also in the probability of mutation and CNV. Out of the 20 SMGs, 11 SMGs exhibited significant mutation differences, six of which exhibited a higher mutation frequency in the high-risk group, including *TTN*, *MUC16*, *CSMD3*, *AXIN1*, and *CACNA1E*, while in the contrary, five of which, *TP53*, *BAP1*, *RYR1*, *MIT1L*, *UNCB0*, and *RYR2* mutated more frequently in the low-risk group (**Figure S4A**). Consistent with the mutation, significant CNV in two phenotypes which might result in distinct biological behaviors in stages I–III HCC also differed. By employing the cBioPortal database, we ultimately identified significant CNV which encompassed the top 10 amplification and deletion genes in the two groups separately (**Figure S4B**). The genes which have been reported to involve the cancer cell invasion (*ENPP2*, *ADCY8*), dysregulated cellular metabolism (*CYC1*), and angiogenesis (*ANGPT1*), immune inhibition (*ANXA3*) amplified notably in the high-risk group, which might elaborate the aggressiveness and malignancy in this group. Moreover, the low-risk group was inclined to maintain more *ZHX1*, *WDYHV1*, *FBXO32*, *ATAD2*, and *PKLR* amplification referring to transcriptional deregulation, TGF-beta1/Smad3 signaling pathway, oxidative phosphorylation, and deletions of tumor suppressor genes *CSMD1*, *ERICH1*, *MYOM2*, and *FBXO25*. In conclusion, the high-risk group exhibits a different genetic pattern from the low-risk group, which can be used to recognize the patients at high risk of LR and then to implement a precision medicine strategy.

### Immune Cell Infiltration in the High- and Low-Risk Groups

The above research indicated that diverse immune status may predominate in two groups, thus CIBERSORT and xCell algorithm were further applied to estimate the infiltration status if immune cells. CD8+ T cells, which implement cytolytic activity to kill tumor cells increased significantly in the low-risk group (**Figure S4C**). Furthermore, we noticed that immune system in the low-risk group performed a host-protecting role, such as increased naïve B cells, activated NK cells, CD8+ T cells, and follicular helper T cells, though there was no statistical significance. Immune cells infiltrating in the high-risk group mainly included naive CD4+ T cells, macrophages (M0), and gamma delta T cells. The distinction was validated by the xCell algorithm; granulocyte macrophage progenitor (GMP) increased in the high-risk group whereas the naive B cells increased in the low-risk group (**Figure S4D**). Studies have characterized the importance of co-stimulating and co-inhibiting molecular functions based on the immune microenvironment. Therefore, we aimed to identify the differences in the expression levels of B7-CD28, TNF superfamily, and other factors between the two groups, and we found the expression of *ICOS* and *TNFRSF4* increases significantly in the low-risk group (**Figure 4C**).

### Assessment of Chemotherapy

Furthermore, by performing pRRophetic R package, we estimated the imputed responses to 138 chemotherapeutic agents among patients in stages I–III HCC to dertermine potential drugs with specific sensitivity to both groups. Eventually, as displayed in **Figure S4E**, a total of seven drugs were identified. One of the seven drugs, BMS.708163 known as a Notch inhibitor was more sensitive to patients in the low-risk group. Of note, the other six of the seven drugs have shown specific sensitivity to patients in the high-risk group. For instance, VX.680, a potent and selective small-molecule inhibitor of the Aurora kinases, was more sensitivity to patients in the high-risk group; BI.2536, a *Plk1* inhibitor, also showed more sensitivity to the high-risk group. Of the seven drugs, a multitude of their targeted pathways were associated with tumor cell prolifiation, such as, cell cycle, chromatin histone acetylation, *PI3K*/*MTOR* signaling, mitosis, and kinases, which were the latent targets of patients in the high-risk group (**Figure 4D**). These results further demonstrated that patients in the high-risk group might have several choices of chemotherapy drugs for prevention and treatment. Our research provided the patients in the high-risk group with a resource for precision chemotherapy and long-term management.

## Discussion

HCC is the fifth most common tumor worldwide and the fourth most common cause of cancer-related deaths in China (14). Although advances in the treatment and management of patients with HCC have improved survival rates to some extent, it still has a high rate of recurrence, limiting long-term survival even after surgical resection. Thus, the deciphering the genomic landscape and recognition of predictive factors for LR could improve patient management and guide precise medication of chemotherapy drugs for patients with LR. For early recurrence, there are some research indicating different signatures can be used as predictors (15), while few data are available for LR.

In the current study, specific mutation landscape in the LR-HCC was revealed comprehensively for the first time. The LR-HCC exhibited different significant mutation genes compared with NR-HCC, which indicated that different driven genes exerted roles in the tumorogenesis and prognosis in two groups of HCC. The top 3 most frequently mutated genes are *TTN* (28%), *CTNNB1* (24%), and *MUC16* (20%). *TTN* encodes a giant protein (>30,000 amino acids) and is rarely recognized as a tumor-associated gene. Recent studies have suggested that TTN mutation is associated with increased TMB and better response to ICIs; however, its role in the development of HCC still needs to be evaluated (16, 17). *CTNNB1* mutations which induce excessive activation of Wnt-β-catenin pathway in HCC play a crucial role in regulating tumor cell proliferation and survival and in tumor angiogenesis (18). *MUC16*, the coding gene of mucin 16, promotes the proliferation and metastasis of cancer cells, and the cancer antigen *CA125*, as an epitope present on mucin 16, is the most commonly used serum biomarker in epithelial ovarian cancer (19). Besides, we found *BAP1* and *RYR1* mutated exclusively in NR-HCC compared with patients with LR. A previous study found patients with *BAP1*-mutation HCC could benefit from drugs inactivating *PKA* and immunomodulators (20), which gave some hints that NR-HCC may benefit from these therapies. Although co-mutations are common in different cancer types and exert various roles in tumorigenesis, especially in lung adenocarcinomas (21), further research is still needed for our discovery to explore the underlying regular mechanism.

In the past years, several scoring systems have beeen developed for estimating HCC early recurrence or overall recurrence (22, 23), while LR was rarelly assessed. In this research, we developed a novel risk score system consisting of *ANGPT4*, *AMFR*, *COLEC12*, *FAM78B*, *LMTK3*, and *TRABD2A*, which has the ability to accurately predict the probability of LR in HCC patients. The reproducibility and powerful performance of six-gene model in multiple independent cohorts and external qRT-PCR data not only prove that it is a robust and highly accurate model but is also promising to be routinely implemented into clinical practice due to the following advantages: high sensitivity and specificity, simplicity, and low cost of qRT-PCR. Among the six genes, *AMFR* and *LMTKS* have been reported to associate with cellular adhesion, invasion, and migration, which may alter the metastatic activity of cancer cells and even play a crucial role as a target for anticancer agents (24–26). In *AMFP*, an internalizing cell surface receptor, upregulation is significantly correlated with more advanced tumor stage and a decreased survival for cancer of the lung, esophagus, stomach, colon, rectum, liver, and skin. *LMTK3*, an oncogenic kinase, promotes tumorogenesis in blader cancer **via* ERK/MAPK* pathway and invasion in breast cancer **via* GRB2*-mediated induction of integrin β_1_ (27, 28). The relationship between the other four genes and migration have never been investigated before, suggesting that these genes may be novel biomarkers in the prognosis of cancer.

Based on the model, we aimed to characterize the function and immune microenvironment of the different groups of cancers. Utilizing GSEA, we found that the high-risk group is enriched in B-cell receptor signaling pathway, focal adhesion, and primary bile acid biosynthesis in KEGG analysis, which consisted of the recurrent features. Besides, the immune cell infiltration in the two groups were explored using CIBERSORT and xCell algorithm, and few differences were identified, such as the CD8+ T cells and naïve B cells are increased in the low-risk group, and naive CD4+ T cells, macrophages (M0), gamma delta T cells, and granulocyte macrophage progenitor (GMP) are increased in the high-risk group. Tumor immune microenvironment play a crucial role, e.g., “soil” in the proliferation, migration, and invasion of cancer cells, which consist of immune cells and other immune-related factors (29). We also found ICOS and TNFRSF4, two well-known co-stimulating molecular functions, increased significantly in the low-risk group. The different immune landscapes in the two groups may contribute to the improvement of prognosis and further selection of immune therapy.

In addition, the different molecular characteristics between the two groups were also uncovered. As the figure displayed, *TTN*, *MUC16*, *CSMD3*, *AXIN1*, and *CACNA1E* mutations characterized the high-risk group. Notably, three of these five genes, *TTN*, *MUC16*, and *AXIN1* were reported in a previously published series as the driver genes in hepatocarcinogenesis (30–32), while *CSMD3* acted as a tumor suppressor gene and decreased expression contributing to hepatocarcinogenesis (33). Of particular interest, *CACNA1E*, the major subunit of the voltage-dependent CaV2.3 Ca2+ channel, may be involved in regulating metabolism-related functions of the liver, including bile secretion, glucose and lipid metabolism, and mitochondria functions, while the specific function of hepatocytes needs more extensive study (34). The low-risk group was characterized by *TP53*, *BAP1*, *RYR1*, *MIT1L*, *UNCB0*, and *RYR2* mutations. Except for *TP53* and *BAP1* which have been reported as oncogenes, the other four genes have not been explored in hepatocarcinogenesis, suggesting that they may be potential tumor-related genes. Besides, CNVs in the two groups were described above. Of note, the high-risk group characterized by more aggressive and malignant phenotype possessed some invasion-related gene amplification (*ENPP2*, *ADCY8*) (35), dysregulated cellular metabolism-related amplification (*CYC1*) (36), angiogenesis-related amplification (*ANGPT1*) (37), and immune inhibition-related amplification (*ANXA3*) (38). Low-risk group maintained *ZHX1*, *WDYHV1*, *FBXO32*, *ATAD2*, and *PKLR* amplifications referring to transcriptional deregulation, TGF-beta1/Smad3 signaling pathway, and oxidative phosphorylation, as well as deletion of tumor suppressor genes *CSMD1*, *ERICH1*, *MYOM2*, and *FBXO25*. Taken together, genomic analysis across the different risk groups of HCC revealed the mechanisms of tumor progression and helped to identify biomarkers in response to targeted therapies.

Subsequently, as a supplement, we verified some potential chemotherapy drugs with specific sensitivity to each group, and most drugs were more sensitive to the high-risk group. These results may offer more therapeutic opportunities for the patients suffering from LR. Therefore, to improve the prognosis of LR-HCC, we should not only strengthen monitoring the patients susceptible to LR but choose suitable antitumor drugs for early prevention and treatment. The present research also have some limitations. First, the utility of the six-gene signature still needs more clinical applications to further validate. Second, we only exhibited the genomic landscape in each group while the reasons behind this phenomenon in terms of specific signaling pathways and molecular mechanisms require further understanding.

## Conclusion

In summary, it is the first time to research the genomic landscape and tumor-infiltrating immune cells in the LR-HCC comprehensively. Using a systematic biomarker discovery and validation approach, we established and validated a stable and powerful six-gene signature for evaluating the LR risk of patients with stages I–III HCC. Our study demonstrated that the LR-related model provides a promising tool to optimize decision-making in surveillance protocol and individual management for patients with stages I–III HCC.

## Data Availability Statement

The original contributions presented in the study are included in the article/**Supplementary Material**. Further inquiries can be directed to the corresponding authors.

## Ethics Statement

The human cancer tissues used in this study were approved by the Ethics Committee of The First Affiliated Hospital of Zhengzhou University in December 19, 2019, and the TRN is 2019-KW-423. The patients/participants provided their written informed consent to participate in this study.

## Author Contributions

YZ and ZLiu designed this work. YZ, ZLi, XL, LL, and LW integrated and analyzed the data. YZ, ZLiu, and XL wrote this manuscript. YZ, ZLiu, ZLi, and XH edited and revised the manuscript. All authors contributed to the article and approved the submitted version.

## Funding

This study was supported by the National Natural Science Foundation of China (Grant No. U1904143) and the National Science and Technology Major Project, Prevention and Treatment of Major Infectious Diseases such as AIDS and Viral Hepatitis (2018ZX10303502).

## Conflict of Interest

The authors declare that the research was conducted in the absence of any commercial or financial relationships that could be construed as a potential conflict of interest.

## Publisher’s Note

All claims expressed in this article are solely those of the authors and do not necessarily represent those of their affiliated organizations, or those of the publisher, the editors and the reviewers. Any product that may be evaluated in this article, or claim that may be made by its manufacturer, is not guaranteed or endorsed by the publisher.

## Supplementary Material

Supplementary Figure 1 Analyses of somatic mutation profiles in HCC samples and enrichment analysis of LR-related genes. **(A)** Summary of the detail mutation information. **(B)** The boxplot displayed the differences of the TMB in two groups. **(C)** Co-occurrence and co-exclusion analysis of the mutated genes in the LR-HCC. **(D)** The dot plots of the GO-enriched analysis based on the LR-relative genes; from left to right: the biology process (BP), cellular component (CC), and molecular function (MF), respectively. **(E)** The hallmark signature from the MSigDB database was utilized to search the tumor-related pathways between two groups.

Supplementary Figure 2 Survival analysis of the six SLRGs and validation of the LR-related signature in the GSE76427 cohort. **(A–F)** The Kaplan-Meier analysis of the six SLRGs. The six SLRGs were *ANGPT4*, *AMFR*, *COLEC12*, *FAM78B*, *LMTK3*, and *TRABD2A*. **(G)** Kaplan-Meier analysis. **(H)** The ROC curve of the model. **(I)** The distribution of risk score, recurrence status, and gene expression panel. **(J**, **K)** The forest plot of the univariate **(J)** and multivariate **(K)** Cox regression analysis for TCGA-LIHC.

Supplementary Figure 3 Validation of six-gene model in a clinical in-house cohort. **(A–F)** Comparison of the six SLRGs in the high- and low-risk groups. **(G)** The distribution of risk score, recurrence status, and gene expression panel.

Supplementary Figure 4 Molecular landscape, immune landscape, and assessment of chemotherapy. **(A)** The significant mutated genes in the two groups. **(B)** The copy number variation in different risk groups. **(C)** The bar plots displayed the proportion of 23 immune cells. **(D)** The proportion of GMP, naive B cells, and NKT in different risk groups. **(E)** The IC50 of BMS.708163 is higher in the high-risk group, whereas the IC_50_ of CMK, GW843682X, JW.7.52.1, MS.275, VX.680, and BI.2536 is higher in the low-risk group.
