# Supplementary figures and images for: Comprehensive Molecular Analyses of a Six-Gene Signature for Predicting Late Recurrence of Hepatocellular Carcinoma
Source: Front Oncol. 2021 Sep 9;11:732447. doi: 10.3389/fonc.2021.732447 (PMC8459683; doi:10.3389/fonc.2021.732447)

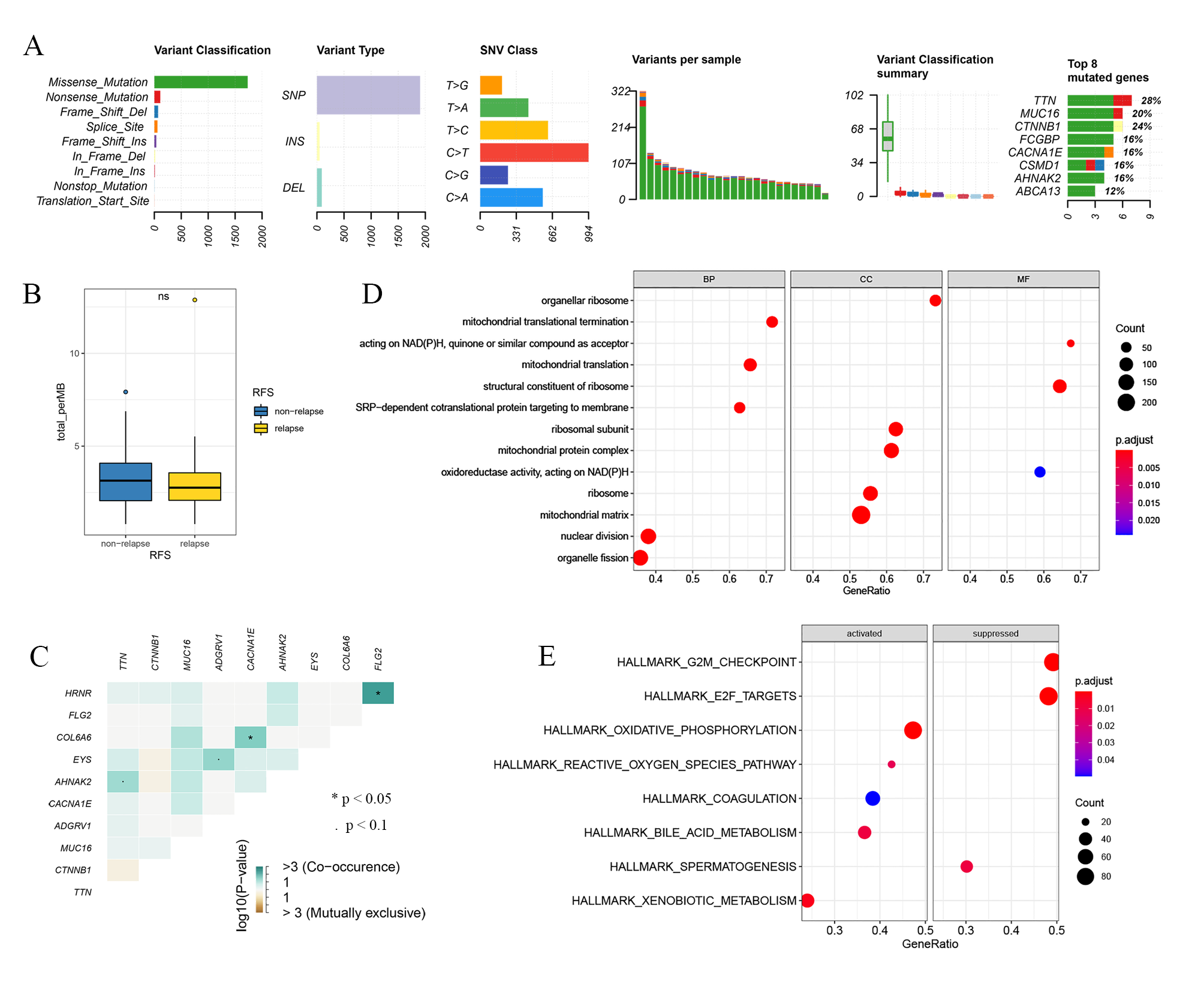

Supplement: Supplementary Figure 1 — Analyses of somatic mutation profiles in HCC samples and enrichment analysis of LR-related genes. (A) Summary of the detail mutation information. (B) The boxplot displayed the differences of the TMB in two groups. (C) Co-occurrence and co-exclusion analysis of the mutated genes in the LR-HCC. (D) The dot plots of the GO-enriched analysis based on the LR-relative genes; from left to right: the biology process (BP), cellular component (CC), and molecular function (MF), respectively. (E) The hallmark signature from the MSigDB database was utilized to search the tumor-related pathways between two groups. [file Image_1.tif]

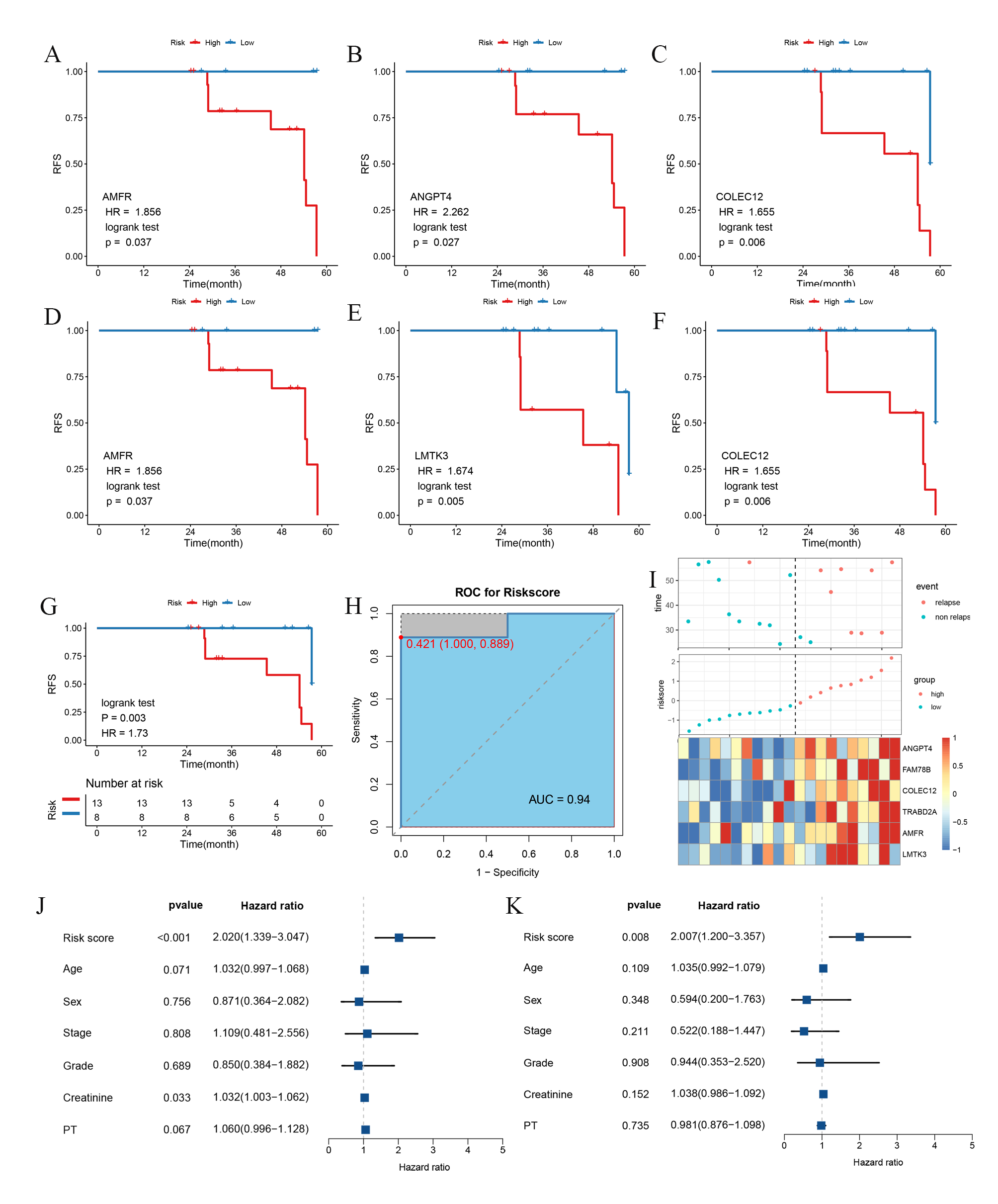

Supplement: Supplementary Figure 2 — Survival analysis of the six SLRGs and validation of the LR-related signature in the GSE76427 cohort. (A–F) The Kaplan-Meier analysis of the six SLRGs. The six SLRGs were ANGPT4, AMFR, COLEC12, FAM78B, LMTK3, and TRABD2A. (G) Kaplan-Meier analysis. (H) The ROC curve of the model. (I) The distribution of risk score, recurrence status, and gene expression panel. (J, K) The forest plot of the univariate (J) and multivariate (K) Cox regression analysis for TCGA-LIHC. [file Image_2.tif]

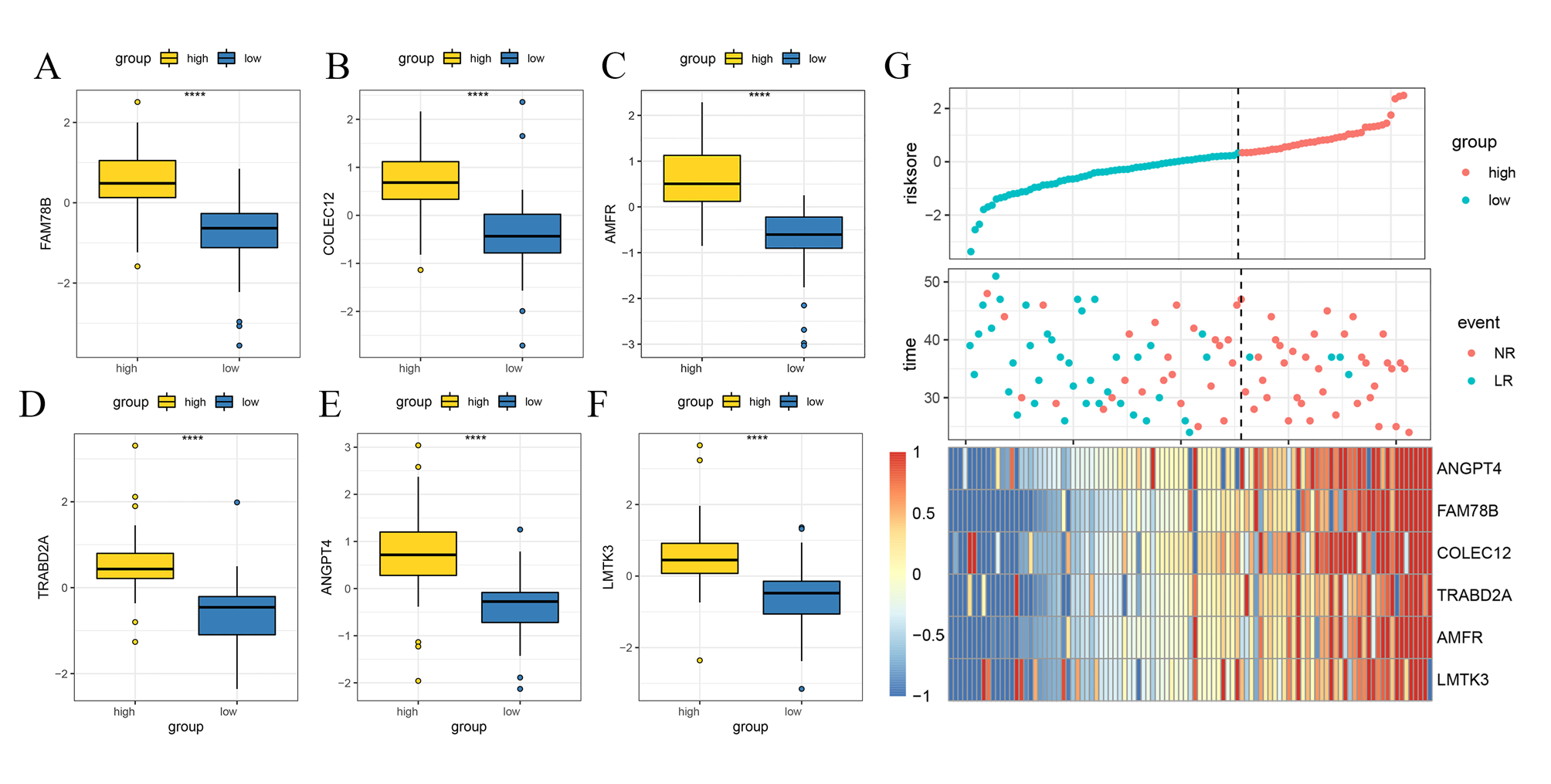

Supplement: Supplementary Figure 3 — Validation of six-gene model in a clinical in-house cohort. (A–F) Comparison of the six SLRGs in the high- and low-risk groups. (G) The distribution of risk score, recurrence status, and gene expression panel. [file Image_3.tif]

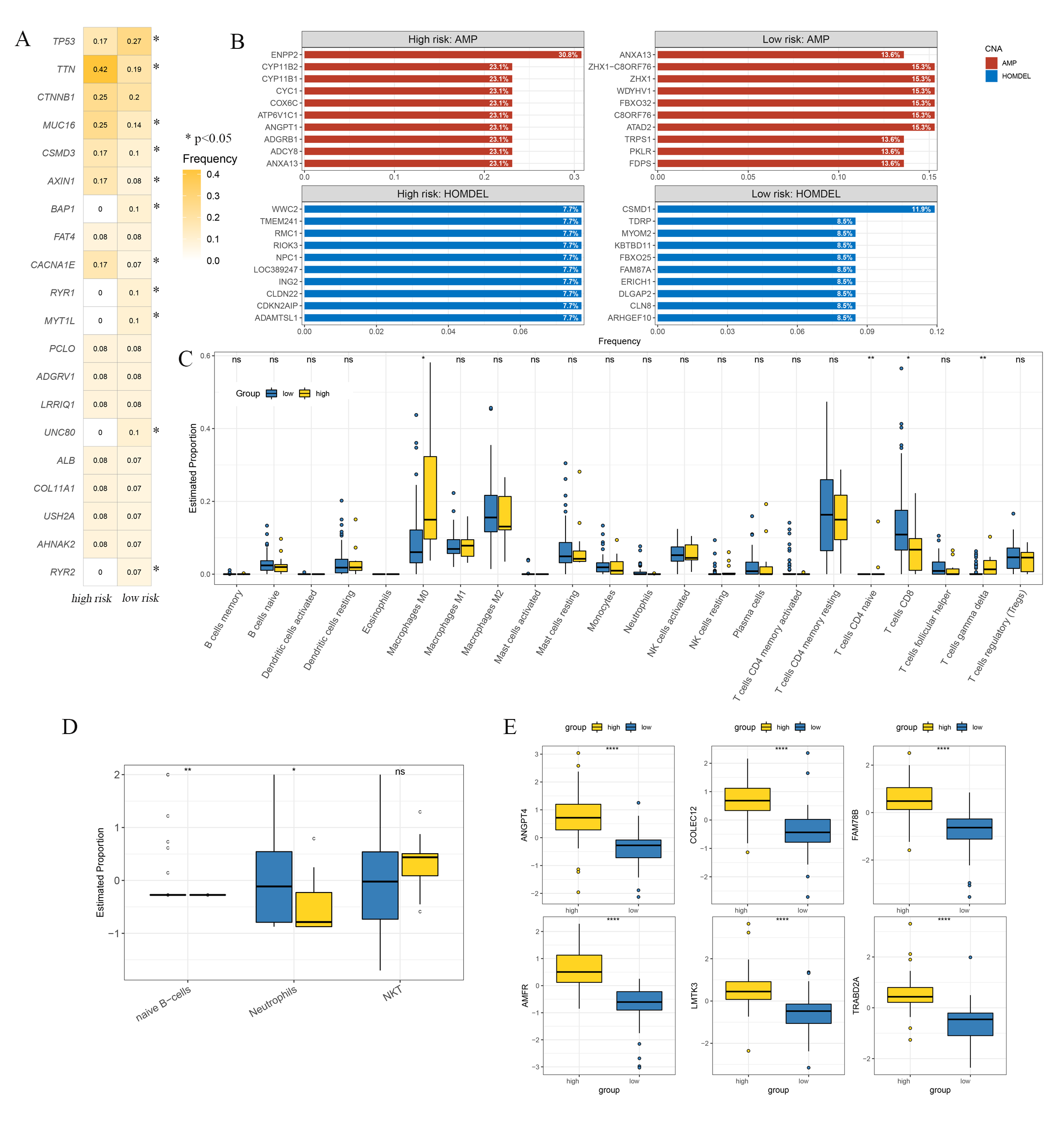

Supplement: Supplementary Figure 4 — Molecular landscape, immune landscape, and assessment of chemotherapy. (A) The significant mutated genes in the two groups. (B) The copy number variation in different risk groups. (C) The bar plots displayed the proportion of 23 immune cells. (D) The proportion of GMP, naive B cells, and NKT in different risk groups. (E) The IC50 of BMS.708163 is higher in the high-risk group, whereas the IC50 of CMK, GW843682X, JW.7.52.1, MS.275, VX.680, and BI.2536 is higher in the low-risk group. [file Image_4.tif]
